# Target-Aware Decoupled Metric-Depth Estimation for Top-View Crane Safety Surveillance

**DOI:** 10.3390/s26185785

**Published:** 2026-09-11

**Authors:** Min Woo Woo, Jaeil Kim, Byeong Hak Kim

**Affiliations:** 1 School of Computer Science and Engineering, IT College, Kyungpook National University, Daegu 41566, Republic of Korea; wmw@kitech.re.kr; 2 Advanced Mobility and Robot System Group, Korea Institute of Industrial Technology, Daegu 42994, Republic of Korea

**Keywords:** crane safety monitoring, industrial sensing, monocular depth estimation, object detection, RGB–LiDAR calibration, target-aware perception, top-view vision

## Abstract

Estimating metric depth for safety-relevant targets from a top-view crane camera is challenging because monocular predictions are scale-ambiguous and target regions of interest (RoIs) mix returns from payloads, personnel, hoisting wires, and the ground. We present a target-focused pipeline in which a detector defines RoIs, a dense metric-depth map is predicted, and one representative depth is extracted per object. A DINOv2–DPT relative-depth network is fixed, while a multiscale adapter, DINO detection branch, and pixel-wise spatial affine calibration head are trained. Ground-truth bounding boxes restrict metric supervision to target-relevant regions but are not inputs to the calibration head. At evaluation, all depth methods use identical predicted RoIs, and a class-aware kernel-density estimation rule extracts representative depths. On 272 hardware-synchronized RGB–LiDAR frames from one operational 150-ton crane, the proposed method achieved a target-level RMSE of 0.564 m and a pixel-level inlier ratio of 0.966 for δ<1.25. One-to-one ground-truth matching yielded a 0.566 m target RMSE with detection precision/recall of 0.960/0.970. AdaBins obtained lower pixel-level MAE and RMSE, whereas the proposed method obtained lower target-level errors. These results demonstrate the feasibility of target-focused metric ranging under the evaluated operational crane conditions.

## 1. Introduction

Industrial sectors such as construction and logistics increasingly use automation and machine perception to support accident prevention [1]. This trend has motivated research on integrating vision-based perception into heavy machinery, including cranes [2]. Monitoring safety-relevant targets within the crane operating area requires 3D information in addition to 2D localization [3,4].

Tracking suspended payloads and ground personnel benefits from estimating their physical distance from the camera [5]. LiDAR can provide this information, but its projected measurements may be sparse on distant targets and its installation introduces sensor, calibration, and maintenance costs [6,7]. We therefore investigate a monocular camera as a complementary target-ranging signal and evaluate it using synchronized measurements from an operational crane installation.

However, monocular depth estimation (MDE) faces scale ambiguity because metric depth must be inferred from a single 2D image [8]. Large-scale models, such as Depth Anything [9], have shown broad zero-shot relative-depth capability. Their training and evaluation, however, largely use conventional perspective views, including autonomous-driving and indoor scenes. Crane-mounted cameras look vertically downward, without a visual horizon, and suspended payloads lack ground-contact cues. These conditions differ from common benchmark settings and can affect metric-scale transfer [10]. In our experiments, Depth Anything V2 [11] and Depth Anything 3 [12] produced pixel-level root mean square errors (RMSEs) of 1.886 m and 10.519 m, respectively, in this crane domain, motivating domain-specific calibration.

Two relevant strategies are global calibration and supervised end-to-end (E2E) metric-depth estimation. Global calibration applies one scale and translation to a relative-depth map [13]. As a GT-assisted diagnostic baseline, we fitted this affine transformation separately to all valid ground-truth (GT) pixels in each test image; the resulting pixel-level RMSE within the evaluated target RoIs was 1.657 m. Supervised models such as ZoeDepth [14] and AdaBins [15] instead learn domain-specific metric distributions. In our data, these models obtained lower average pixel-level errors but higher target-level scalar errors than the proposed method. This metric-dependent contrast motivates a pipeline that is optimized and evaluated around the target-depth quantity required by the application.

**Figure 1:**
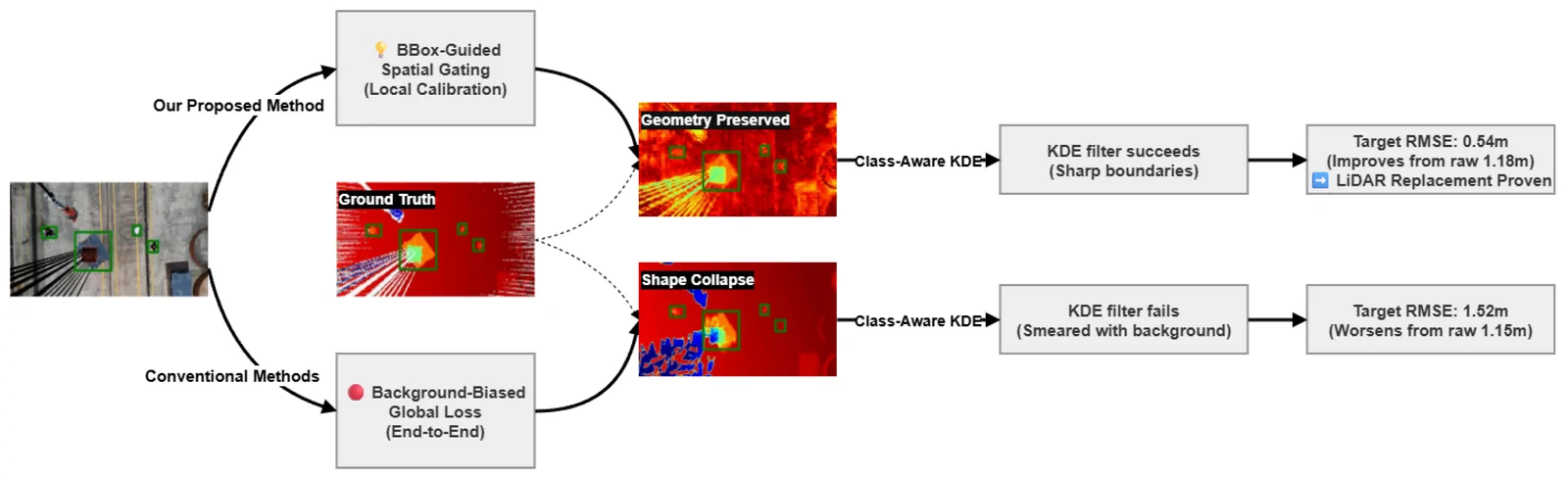
Conceptual comparison of global end-to-end depth regression and the proposed target-focused pipeline. GT BBoxes restrict metric supervision during training, whereas predicted RoIs support target-distance extraction during inference. Green boxes denote target RoIs, solid arrows indicate the processing flow, and dashed arrows link the GT scene to the representative depth outcomes.

Figure 1 provides motivation for a target-focused alternative. Instead of requiring uniform accuracy over the complete image, we optimize metric calibration only where the application requests a measurement. KDE [16] is subsequently used to extract a representative value from an RoI that may contain several depth layers.

The main contribution is an application-oriented sensing pipeline for local metric-depth estimation within detected target RoIs in a top-view crane environment. The pipeline separates fixed relative-depth extraction from trainable metric calibration. A DINO detection branch and a pixel-wise spatial affine calibration head use features produced through the trainable multiscale adapter from the fixed DINOv2 backbone. During training, GT bounding boxes (BBoxes) define the support of the metric loss, thereby increasing the relative contribution of target RoI pixels. The BBoxes do not directly condition the calibration head, and the resulting depth map is dense; “target-aware” therefore refers to the spatial support of supervision and evaluation. At inference, predicted BBoxes define the regions of interest (RoIs) from which target-depth estimates are extracted. Under this protocol, the pipeline achieved a target-level RMSE of 0.564 m.

The primary contributions of this paper can be summarized as follows:

- **Industrial task and measurement definition:** We formulate target-focused monocular depth estimation for suspended payloads and ground personnel, distinguishing RoI pixel-level depth accuracy from the error of one representative RoI depth value per detected target.
- **Target-focused lightweight adaptation:** We retain a fixed relative-depth network and jointly train a multiscale adapter, a DINO detection branch, and a pixel-wise spatial affine calibration head. GT BBox masks restrict metric supervision to target-relevant regions without serving as differentiable inputs to the detector or calibration head.
- **Operational multimodal dataset and application-aligned evaluation:** We acquire hardware-synchronized and geometrically calibrated RGB–LiDAR observations from an operational 150-ton crawler crane with suspended payloads and ground personnel. We compare all evaluated depth methods using identical predicted RoIs and report both pixel-level and KDE-based target-level metrics. The two evaluations produce different method rankings, motivating application-aligned reporting.
- **Detection-matched sensitivity analysis:** We additionally match detections one-to-one to class-consistent GT boxes at IoU ≥0.5. The resulting target-level RMSE differs by 0.002 m from the common-RoI result.

## 2. Related Work

### 2.1. Monocular Depth Estimation and Foundation Models

Early research in MDE primarily focused on pixel-wise regression using convolutional neural networks (CNNs) [17]. With the advent of vision transformers, DPT [18] improved global-context modeling for dense prediction. More recently, Depth Anything [9] has used large mixed datasets to improve zero-shot relative-depth generalization. Its DINOv2 encoder [19] provides self-supervised visual representations, while Depth Anything V2 [11] combines these features with a dense decoder. Training across heterogeneous cameras and scales commonly emphasizes affine-invariant relative depth [20]; consequently, a domain-specific metric relation is still required. This issue is pronounced for the top-down crane views considered here, where a visual horizon and conventional ground-contact cues are absent.

### 2.2. Metric-Depth Estimation and Domain Adaptation

To overcome relative-depth scale ambiguity, AdaBins [15] predicts adaptive metric bins, while ZoeDepth [14] combines relative- and metric-depth components. When adapting a large model to a compact target-domain dataset, preserving pretrained representations can support stable transfer [21]. Separately, a global pixel objective weights samples according to their frequency; in a ground-dominated crane image, this may under-represent the application targets. These considerations motivate our fixed-foundation, target-focused design.

### 2.3. Task Affinity and Parameter-Efficient Adaptation

Multi-task and transfer learning studies have shown useful affinity between detection, semantics, and geometry [22,23,24], while also documenting possible gradient interference [25]. Parameter-efficient adaptation [26] and frozen-backbone designs can reduce the number of updated parameters. Our design uses this principle but differs in its supervision support: the metric loss is evaluated only over valid depth samples inside annotated target BBoxes.

## 3. Proposed Sensing Pipeline

### 3.1. Motivation and Pipeline Overview

In top-view crane monitoring, the application-relevant outputs are the metric depths of detected personnel and suspended payloads rather than uniformly accurate depth over the entire image. Conventional dense metric-depth training, however, minimizes error over all valid pixels. Because projected LiDAR returns from the ground can substantially outnumber those from the targets, the global objective may assign limited relative weight to the regions that require a measurement. This mismatch motivates a target-focused pipeline that retains dense depth prediction while concentrating metric supervision on annotated target RoIs.

**Figure 2:**
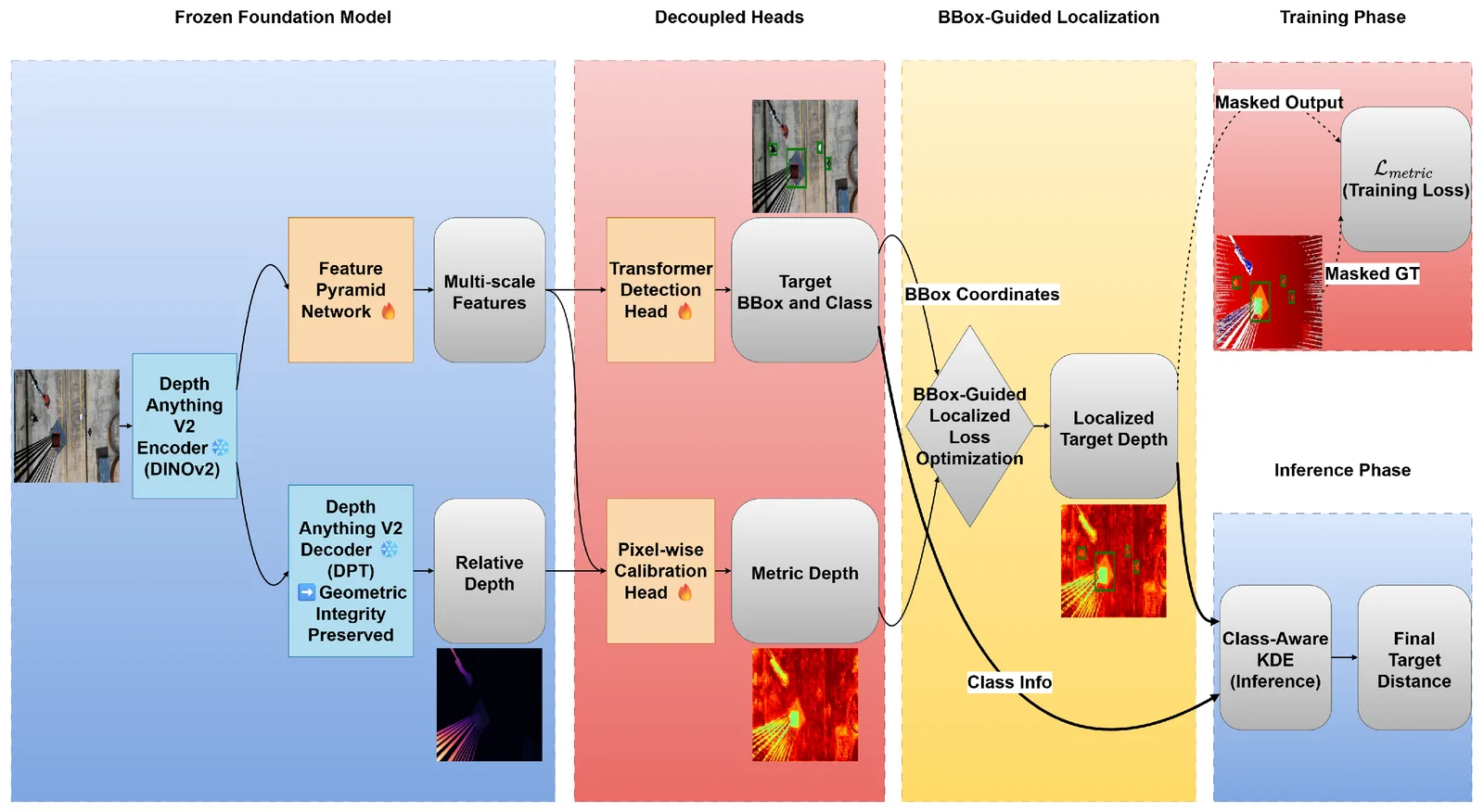
Overview of the proposed sensing pipeline. Blue modules are fixed, red modules are trainable, the yellow module denotes GT-BBox-constrained metric supervision, and green boxes denote target BBoxes.

To preserve the general visual representation learned by the foundation model while adapting to the crane domain, we retain the pretrained relative-depth network and learn the components required for target detection and metric calibration [27]. To address these considerations, we use a target-focused, decoupled metric-depth pipeline. As shown in Figure 2, it separates relative-depth extraction from trainable detection and metric calibration.

- **Fixed relative-depth foundation:** A DINOv2 encoder [19] and DPT decoder [18] provide relative depth and shared features. Their pretrained parameters are not updated in the reported model.
- **DINO detection branch:** The trainable DINO detection branch [28], denoted by the fire icon in Figure 2, uses the multiscale adapted features to localize the two target classes.
- **Pixel-wise spatial affine calibration head:** This trainable head predicts spatial affine maps (α,β). It produces a dense output and is made target-aware through the GT-BBox support of its training loss, not through an RoI input.

This separation limits target-domain optimization to the multiscale adapter, DINO detection branch, and calibration head while leaving the relative-depth network fixed.

### 3.2. Fixed Foundation Features

In top-down crane imagery, target surfaces and their boundaries must be distinguished from the ground and foreground wires. We use a fixed DINOv2 foundation backbone [19] and its pretrained DPT decoder as the source of relative depth and image features. The reported configuration keeps these foundation parameters fixed. The network produces a relative-depth map and reusable backbone features, while the calibration head adapts the output through its pixel-wise spatial affine maps. Boundary behavior is evaluated as a property of the complete pipeline.

### 3.3. DINO Detection Branch and Joint Training

The DINO detection branch and calibration head are trained jointly while the Depth Anything V2 encoder and DPT decoder remain fixed. Reusing fixed features for both trainable components is motivated by prior evidence that visual tasks can benefit from shared representations [22,23]. The fixed DINOv2 features provide the shared representation used to train the target detector and metric calibration head on the present dataset.

The frozen DINOv2 backbone outputs patch tokens at a single spatial resolution, whereas the detector operates on a multiscale feature pyramid to accommodate variations in target size. We therefore use a trainable multiscale feature-pyramid adapter based on FPN [29], as shown in Figure 2. The adapter projects the top-layer backbone features using 1×1 convolutions and generates feature maps at four spatial scales. Transposed convolutions increase the resolution by factors of two and four, and max pooling produces a feature map at half the input resolution. The resulting four feature maps are supplied to the detection head.

Table 1 summarizes the feature flow and trainable layers for an 896×896 input. The ViT-L/14 patch grid is 64×64, and the highest-resolution adapted feature is also supplied to the calibration head.

Following DINO [28], the detection head uses two-stage query initialization with 900 object queries and iteratively updates the predicted BBoxes across decoder layers. The DINO detection branch and calibration head are optimized in the same training procedure. Their combined objective is

(1) $$L_{\mathrm{total}}=L_{\det}\left(\hat{Y},Y\right)+\lambda L_{\mathrm{metric}}\left(d_{\mathrm{abs}},d_{\mathrm{gt}}\right)$$

where $\hat{Y}$ denotes the predicted class scores and BBoxes, Y denotes the corresponding GT labels and BBoxes, $L_{\det}$ comprises the focal [30], $\ell_{1}$, and generalized IoU (GIoU) terms used by DINO, and $L_{\mathrm{metric}}$ is the target-focused calibration loss. Both branches use features derived from the same fixed backbone, but their supervision is separate: $L_{\det}$ uses the detector predictions and $L_{\mathrm{metric}}$ uses a hard mask constructed from GT BBoxes. Therefore, we do not assume a differentiable metric-loss path through predicted boxes to the detector.

### 3.4. Pixel-Wise Spatial Affine Calibration Head

To transform the dense relative-depth map $d_{\mathrm{rel}}$ into a dense metric prediction $d_{\mathrm{abs}}$, we use a lightweight calibration head $H_{\phi }$. Target restriction is applied to its loss, not to $d_{\mathrm{rel}}$ or the head input. Unlike global calibration with one image-level scale and offset, the proposed head predicts spatially varying affine parameters. The high-resolution backbone feature map *F* and the spatially aligned relative-depth map are concatenated along the channel dimension and supplied to the calibration head. Let ${\tilde{d}}_{\mathrm{rel}}$ denote $d_{\mathrm{rel}}$ bilinearly resized to the $H_{c}\times W_{c}$ spatial resolution of *F*. The head $H_{\phi }$ consists of a 3×3 convolution followed by ReLU and a 1×1 output convolution. It predicts affine parameter maps $\alpha ,\beta \in R^{1\times H_{c}\times W_{c}}$:

(2) $$\left[\hat{\alpha },\beta \right]=H_{\phi }\left(\mathrm{Concat}(F,{\tilde{d}}_{\mathrm{rel}})\right)$$

To enforce a positive multiplicative scale and support training stability, a Softplus activation is applied to $\hat{\alpha }$:

(3) $$\alpha \left(p\right)=\ln\left(1+\exp(\hat{\alpha }\left(p\right))\right)+\epsilon$$

where $\epsilon =10^{-4}$ and *p* denotes a pixel location at the calibration resolution. The affine output and final dense prediction are

(4) $${\tilde{d}}_{\mathrm{abs}}\left(p\right)=\alpha \left(p\right){\tilde{d}}_{\mathrm{rel}}\left(p\right)+\beta \left(p\right),\;d_{\mathrm{abs}}=U\left({\tilde{d}}_{\mathrm{abs}}\right),$$

where U denotes bilinear resizing to the network input resolution. The positivity constraint on α preserves a positive scale, while the unconstrained β permits spatial offset correction. For numerical stability, final depth values are lower-bounded at $10^{-3}$ m for pixel-level evaluation, and KDE extraction uses the positive RoI values. Gradient descent updates the calibration head while preserving the fixed relative-depth network.

### 3.5. GT-BBox-Guided RoI Loss

In the collected top-down views, projected LiDAR samples from the ground outnumber samples from personnel and suspended payloads, and the ground and target surfaces can be separated by more than 10 m along the depth axis. If the metric loss is applied over the complete valid depth image, ground returns dominate the number of supervised samples. We instead construct a hard spatial mask from the annotated GT BBoxes during training. Importantly, these boxes are not produced by the detector and no gradient passes through the mask construction.

A 2D target RoI support mask $M_{\mathrm{roi}}\left(p\right)$ is defined as

(5) $$M_{\mathrm{roi}}\left(p\right)=\left\{\begin{array}{cc} 1, & \mathrm{if}p\in {⋃}_{k=1}^{K}b_{k}^{gt}\\ 0, & \mathrm{otherwise}. \end{array}\right.$$

The valid supervision set is

(6) $$\Omega =\left\{p|M_{\mathrm{roi}}\left(p\right)=1,d_{\mathrm{gt}}\left(p\right)>0\right\},\;N_{\mathrm{valid}}=\left|\Omega \right|,$$

which retains valid projected LiDAR samples inside the annotated BBoxes. The metric loss is

(7) $$L_{\mathrm{metric}}=\frac{1}{N_{\mathrm{valid}}}\sum_{p\in \Omega }\mathrm{SmoothL1}\left(d_{\mathrm{abs}}\left(p\right),d_{\mathrm{gt}}\left(p\right)\right).$$

Training samples with $N_{\mathrm{valid}}=0$ contribute zero metric loss. The BBox mask provides an annotation-efficient spatial support that increases the contribution of target-adjacent LiDAR samples while preserving contextual returns from object boundaries, hoisting wires, and nearby ground. Thus, $L_{\mathrm{metric}}$ implements RoI-focused supervision using the same annotations available for detector training. Instance masks offer a possible extension for studying more selective target-surface supervision.

### 3.6. Class-Aware KDE-Based Target-Depth Extraction

**Figure 3:**
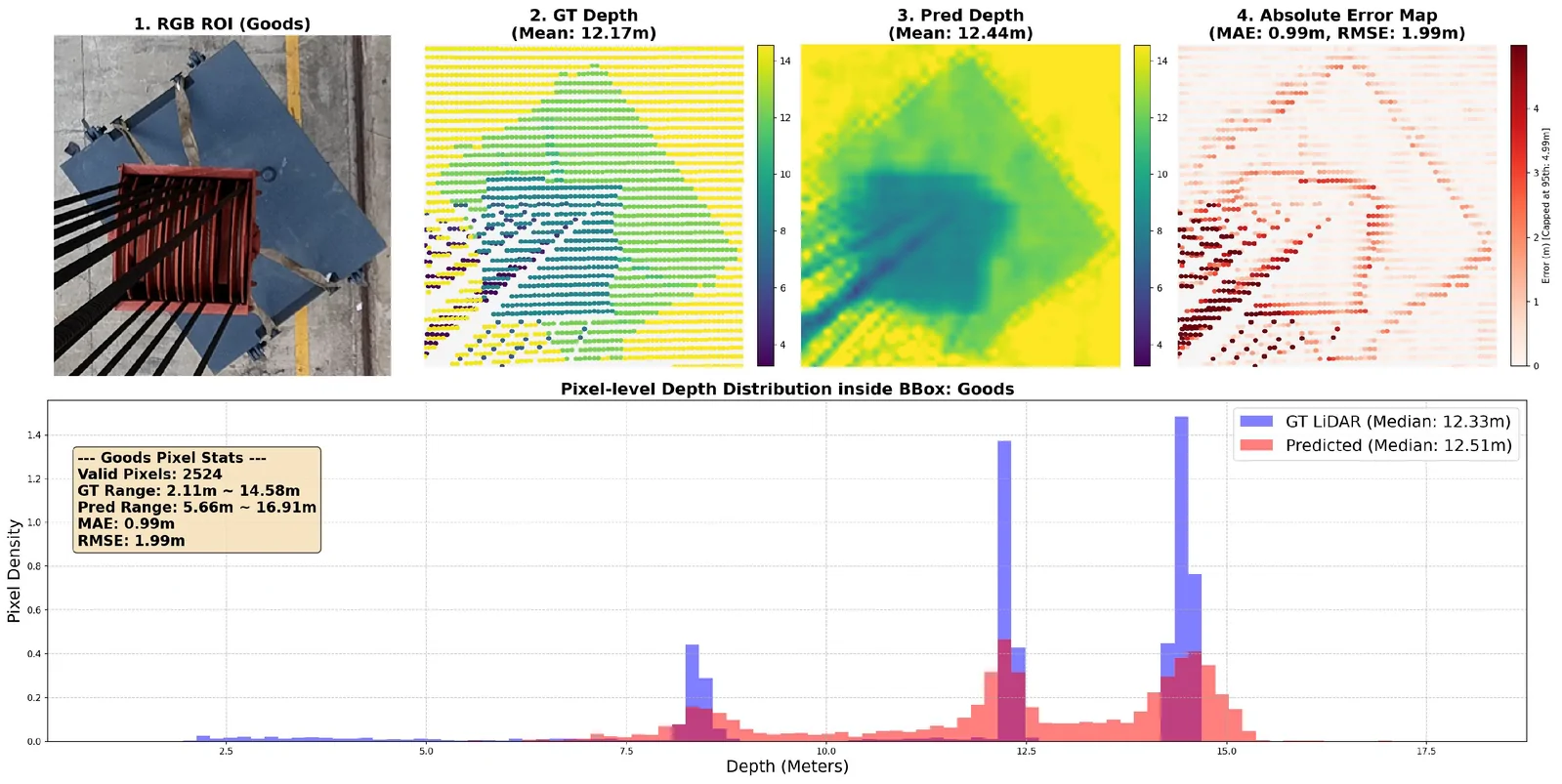
Multimodal depth distribution within a Goods RoI containing wire, payload, and ground returns.

As illustrated in Figure 3, a target RoI can contain multiple depth modes associated with hoisting wires, the target, and the background. To summarize this spatial mixture as one target-depth estimate, we apply class-aware KDE [16], as shown in Figure 2. The probability density function is estimated from the valid depth distribution within the BBox using a Gaussian kernel. Here, “multimodal” denotes a depth distribution with multiple local maxima caused by physical layers such as foreground wires, target objects, and the ground.

We use SciPy’s Gaussian KDE with its default Scott bandwidth factor, $n^{-1/(d+4)}$ for d=1, applied to the sample covariance. The density is evaluated at 100 equally spaced depths between the minimum and maximum valid RoI values. Peaks are detected with a density-prominence threshold of 0.02. The median provides a deterministic fallback when fewer than 50 valid values are available or a valid KDE peak is unavailable. The same exact-KDE implementation is applied to prediction and GT when forming each reported target-level pair.

- Human (Label 1): The rule initially prioritizes the peak closest to the camera, which is intended to represent the upper surface of the person (e.g., a helmet). If three or more peaks are detected, the closest peak is treated as a possible return from an overhanging hoist rope and the second-closest peak is selected.
- Goods (Label 0): With three or more detected peaks, the second closest peak is selected. With two peaks, the peak with the greatest density is selected; with one peak, that peak is returned.

This domain-specific routing rule is evaluated against class-agnostic KDE and center-crop extraction in Section 4.7.

## 4. Experiments and Results

### 4.1. Experimental Setup, Dataset Configuration, and Implementation Details

**Figure 4:**
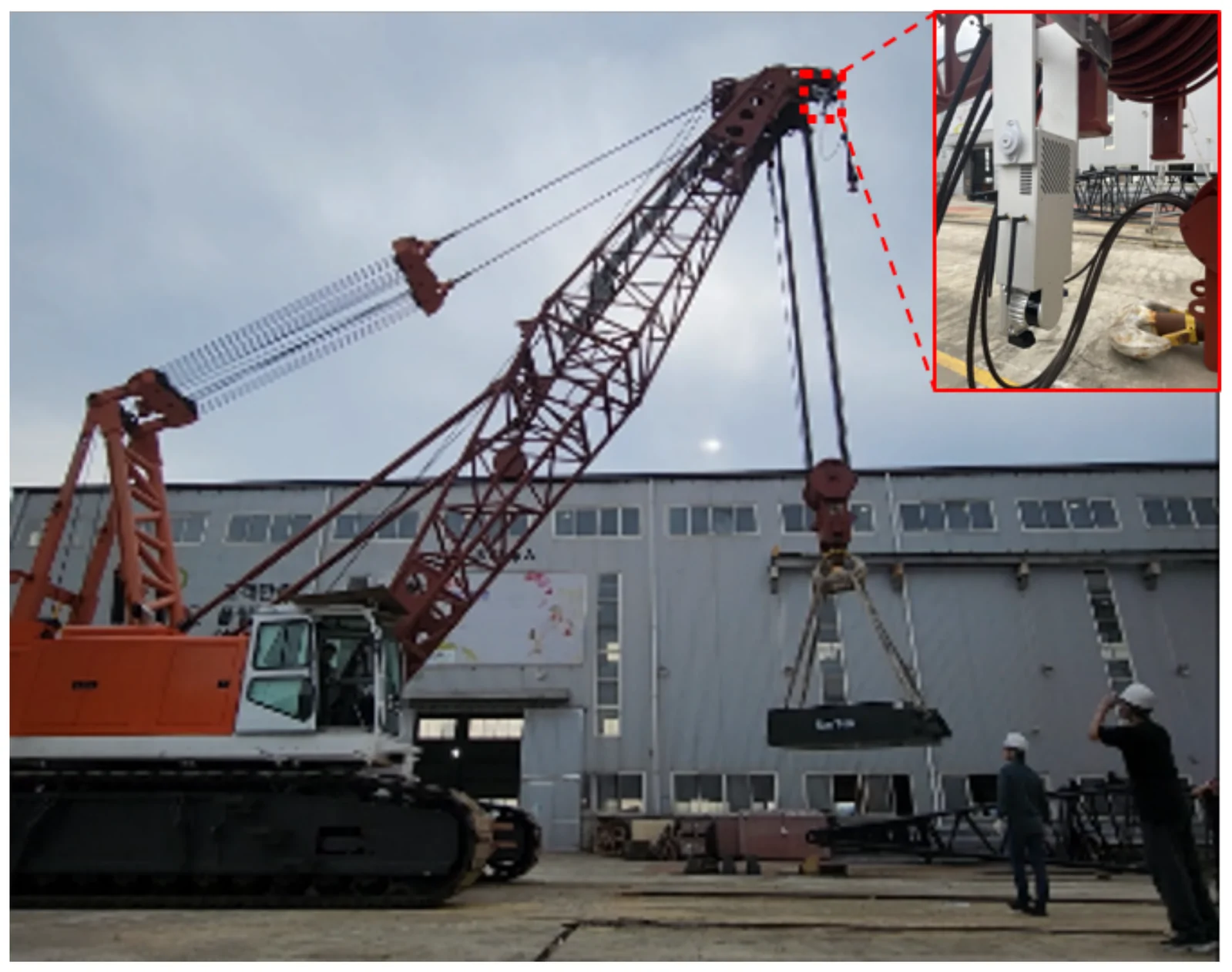
Crawler-crane environment used for data acquisition. The red dashed callout identifies the integrated RGB–LiDAR sensor assembly.

To represent the spatial dynamics and vertical-scale variations of a heavy-machinery environment, we collected a custom multimodal dataset using an operational 150-ton crawler crane in the actual operating environment shown in Figure 4. The red dashed callout identifies the crane-mounted, integrated RGB–LiDAR sensor assembly used for data acquisition. The sensor payload comprised a 128-channel Ouster OS1 LiDAR (Ouster, Inc., San Francisco, CA, USA) and a Teledyne FLIR Blackfly S industrial machine-vision camera (Teledyne FLIR, LLC, Wilsonville, OR, USA). The Ouster OS1 acquired high-density 3D measurements, while the Blackfly S captured hardware-synchronized 4K RGB images.

A cross-modal calibration procedure was used to project LiDAR measurements into the image. Camera intrinsics were estimated with an equidistant distortion model for the wide-field-of-view lens. For extrinsic calibration ($x_{\mathrm{cam}}=Rx_{\mathrm{lidar}}+t$), random sample consensus (RANSAC)-based plane fitting was applied to LiDAR returns from a calibration board. The fitted plane was used when deriving the 3D-to-2D projection to reduce the effect of depth quantization on the calibration correspondences. In the adopted coordinate convention, the X-axis represents metric depth from the camera optical center and the Z-axis represents height normal to the ground plane.

Acquiring synchronized multimodal observations from this platform requires access to operational heavy machinery while maintaining hardware synchronization, cross-modal calibration, and the elevated top-view sensor geometry. The resulting dataset provides sensor-aligned observations of suspended payloads and personnel during actual crane operation and enables evaluation of the proposed target-ranging pipeline under representative top-view motion and scale variation.

Table 2 states the operational scope of the dataset. It contains 272 hardware-synchronized frames (190/55/27 for training/validation/test) and 989 annotated instances (687/203/99) from one crane sensor configuration. The two classes are ground personnel (Human) and suspended payloads (Goods). The train/validation/test subsets contain 101/31/15 straight-travel frames and 89/24/12 slewing frames, respectively.

The primary split was constructed at the frame level while preserving both operating conditions across the training, validation, and test subsets. It supports the common-RoI comparison, while the complementary operating-condition holdout in Section 4.5 evaluates transfer with all slewing frames excluded from training. The camera installation altitude ranged from 15.24 to 16.49 m, and data were collected under daylight and clear-weather operating conditions. Release of a representative anonymized subset will be considered upon publication, subject to applicable data-sharing and institutional requirements, and broader environmental and cross-installation evaluation forms the next validation stage.

The network was implemented in MMDetection (v3.3.0). The KDE analysis used SciPy (v1.13.1), and model training used PyTorch (v2.1.2). Each geometric augmentation was applied consistently to the RGB image, LiDAR depth map, and BBox annotations. Training used aspect-ratio-preserving random resizing, padding and cropping to 896×896, and random horizontal flipping; testing used aspect-ratio-preserving resizing and padding to 896×896. Depth Anything V2-Large supplied the fixed DINOv2 encoder and DPT decoder. The multiscale adapter, DINO detection branch, and pixel-wise spatial affine calibration head were optimized with AdamW for 24 epochs, batch size 1, initial learning rate $10^{-4}$, weight decay $10^{-4}$, and gradient clipping at 0.1. A 500-iteration linear warm-up preceded learning-rate reductions by 0.1 at epochs 11 and 22. The coefficient λ=0.1 was selected empirically in preliminary experiments to provide a practical balance between the metric-depth term and the multi-term DINO objective and was then fixed across all reported runs.

Table 3 provides the archived final-refinement settings of the two supervised baselines. Both were trained on the same 190 RGB–LiDAR training pairs with valid-LiDAR-pixel supervision and deterministic resizing. Each baseline retains the optimization configuration appropriate to its native architecture, while the shared training data and common predicted RoIs provide consistent application-level evaluation support.

### 4.2. Target-Focused RoI Evaluation Protocol

We report two conditional depth evaluations: pixel-level error over valid projected LiDAR samples in each RoI and target-level error for one representative scalar per evaluated RoI. The pixel-level metrics are first computed within each RoI and then macro-averaged across the evaluated RoIs. At test time, BBoxes and class labels are generated by the proposed detector with a confidence threshold of 0.35; GT BBoxes are not used to define the evaluation RoIs.

The same predicted BBoxes and labels are applied to every evaluated depth method, providing common spatial support for a controlled comparison of depth accuracy. The confidence threshold of 0.35 was selected empirically in preliminary experiments as a high-recall operating point appropriate for safety-oriented target ranging and was then fixed for all the reported evaluations. It yielded 0.970 recall in the class-consistent matching analysis. Detector performance and its contribution to target-level depth are reported separately in Section 4.6.

The target-focused evaluation intentionally retains the mixture of target, wire, and background pixels within each BBox, thereby evaluating the downstream extraction rule under the RoI composition encountered at inference.

For each predicted RoI, the class-aware KDE rule in Section 3.6 is applied separately to the predicted depth values and to the projected LiDAR GT values inside the same box. The resulting pair defines the predicted and reference representative RoI-depth values used as target-depth estimates. Pairs for which either side has no positive valid value are excluded. Mean absolute error (MAE), RMSE, and absolute relative error (Abs Rel) are then computed over the collected pairs. Thus, target-level GT is a KDE-derived RoI statistic, not a manually segmented object-surface distance.

**Figure 5:**
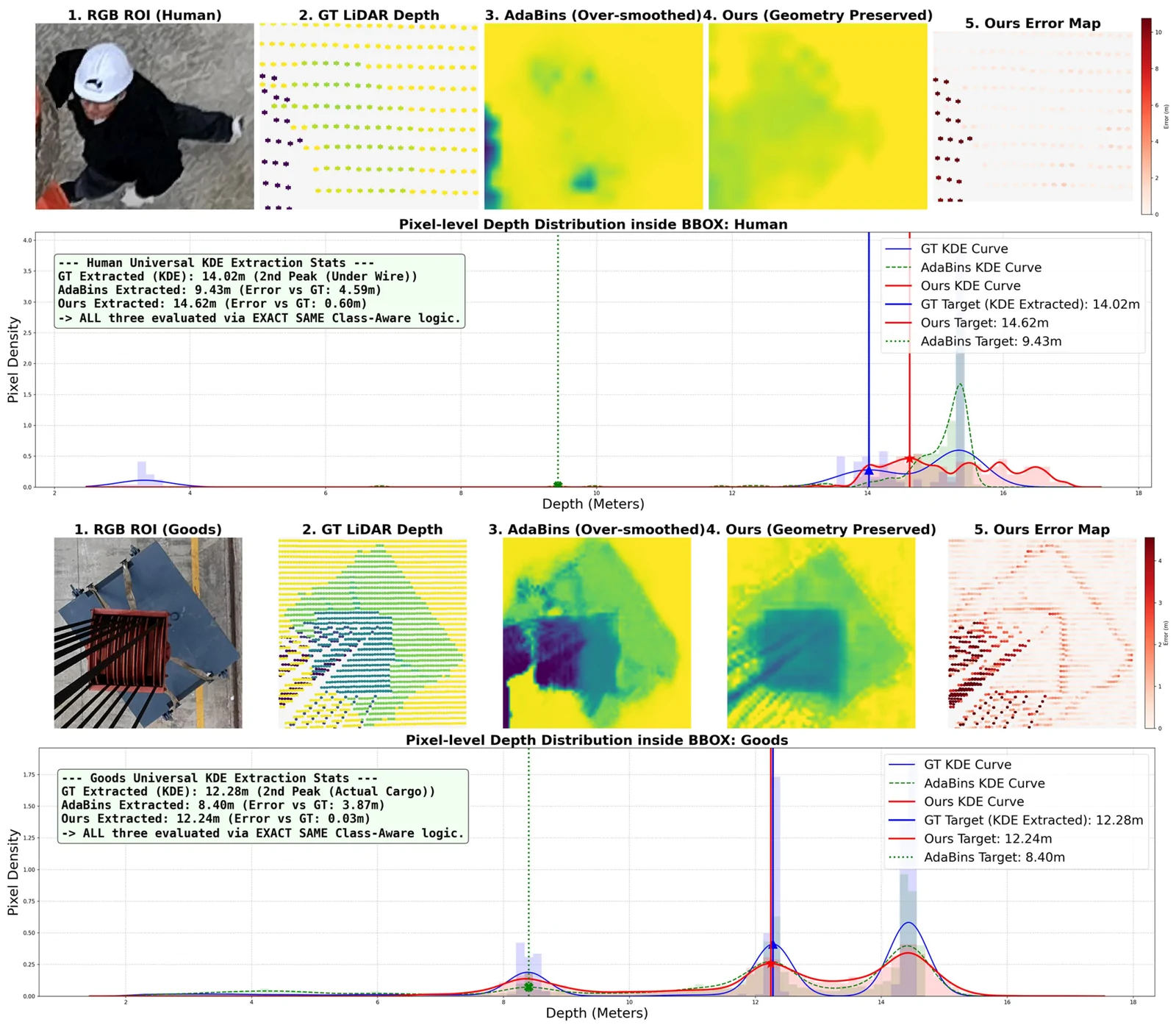
Representative Human and Goods RoIs, depth maps, error maps, and KDE distributions for GT, AdaBins, and the proposed method. Cross-shaped points denote projected LiDAR samples. The colored curves represent the GT, AdaBins, and proposed depth distributions; vertical lines and their associated markers denote the extracted target depths, as identified in the legend. Aggregate performance is reported in Table 4.

Figure 5 visualizes representative RoIs, their depth and error maps, and the corresponding multimodal KDE distributions used by this evaluation protocol.

The quantitative results under the common predicted-RoI protocol are summarized in Table 4 and analyzed in Section 4.3.

### 4.3. Quantitative Performance Evaluation: Metric-Dependent Ranking

Table 4 compares the proposed pipeline with the evaluated zero-shot foundation, test-GT affine, and supervised E2E baselines: Depth Anything V2-Large, Depth Anything 3-Large, ZoeDepth-N, and AdaBins. Pixel-level metrics are computed over valid LiDAR pixels inside each common predicted RoI and macro-averaged across RoIs, whereas target-level metrics compare one class-aware KDE value extracted from prediction and GT for each evaluated RoI. Bold values indicate the best result among the evaluated methods for each metric.

Depth Anything 3-Large [12] and V2-Large [11] recorded pixel-level RMSEs of 10.519 and 1.886 m, respectively. Their zero-shot outputs were evaluated directly without GT-based scale alignment, measuring their metric-scale transfer to the top-view crane domain. The per-image affine diagnostic fits one scale and offset to the fixed Depth Anything V2 output using all valid GT pixels in each test image. It provides a GT-assisted reference for image-specific affine alignment and obtained pixel- and target-level RMSEs of 1.657 and 0.996 m, respectively.

As Table 4 shows, the two metric levels ranked the methods differently. AdaBins recorded lower pixel-level MAE and RMSE (0.604 and 1.151 m) than the proposed method (0.796 and 1.185 m), whereas its errors were higher after target-level extraction. The proposed method obtained the lowest target-level errors and the highest pixel-level inlier ratios. This metric-dependent reversal shows that low average RoI pixel error does not necessarily imply low error in the representative object distance required by the application, supporting the proposed target-level evaluation.

After the same class-aware KDE rule was applied to the prediction and GT, AdaBins yielded target-level MAE and RMSE values of 1.105 and 1.539 m, respectively. The proposed method achieved target-level MAE and RMSE values of 0.455 and 0.564 m and a pixel-level δ<1.25 ratio of 0.966. Together, these results show strong representative target-distance accuracy and high pixel-level inlier performance under the common-RoI protocol.

Because the 100 evaluated predicted RoIs are clustered within 27 test frames, we quantified uncertainty using a frame-cluster bootstrap with 10,000 resamples. All RoIs from a sampled frame were resampled together. As shown in Table 5, the proposed RMSE interval did not overlap the AdaBins interval, providing statistical support for the target-level advantage under the common-RoI evaluation protocol.

The proposed method obtained consistent target-level RMSEs for straight-travel and slewing test frames (0.565 and 0.562 m, respectively), whereas AdaBins obtained 1.484 and 1.614 m. This breakdown demonstrates stable performance across the two operating conditions represented in the primary evaluation. Human RoIs constitute the more demanding target class because of their smaller average image area, while the proposed method retained lower Human and Goods errors than AdaBins.

### 4.4. Isolated Metric-Loss-Support Ablation

To isolate the effect of GT-BBox-supported metric supervision, we trained the same architecture with the metric loss evaluated over all valid projected LiDAR samples. The seed, 24-epoch schedule, optimizer, augmentations, and frozen/trainable modules were unchanged; only the metric-loss support differed. Both depth outputs were evaluated using the same 100 RoIs generated by the reported detector, preventing a change in detected regions from confounding the depth comparison. Table 6 reports this controlled comparison.

As shown in Table 6, RoI-focused loss support reduced target-level RMSE from 0.715 to 0.564 m and pixel-level RMSE within the fixed RoIs from 1.611 to 1.185 m. This controlled comparison directly supports the target-focused supervision strategy used by the proposed pipeline.

### 4.5. Straight-Travel-to-Slewing Holdout

To complement the primary comparison with an independent operating-condition test, we trained the model from scratch on 110 straight-travel frames. A separate 27-frame straight-travel segment was used for validation, with 10 intervening frames excluded as a temporal buffer. All 125 slewing frames were reserved for testing. The architecture, seed, optimizer, augmentations, and epoch schedule were held constant. This protocol evaluates straight-travel-to-slewing transfer within the operational crane installation, with the results reported in Table 7.

As shown in Table 7, the model trained without slewing frames achieved a target-level RMSE of 0.915 m over all 394 held-out GT instances, with a frame-cluster 95% CI of [0.830, 1.003] m. The complete predicted-RoI path achieved 0.610 COCO mAP, 0.915 AP50, and a target-level RMSE of 1.175 m over 520 predicted RoIs at the fixed 0.35 threshold. The difference between the GT- and predicted-RoI results includes detection errors and differences in RoI composition in addition to depth error. This experiment demonstrates target ranging across 125 frames from a physically distinct crane operating condition that was excluded from training, thereby extending the evaluation beyond the primary frame-level split.

### 4.6. GT-Matched Detection Sensitivity Analysis

To quantify the contribution of detector localization to the common-RoI result, we performed an additional class-consistent matching analysis. Predicted and GT boxes were paired one-to-one within each image and class by descending IoU, accepting pairs at IoU ≥0.5. Depth metrics were recomputed for the resulting matched true positives, while unmatched predictions and GT boxes were included in the detection statistics as false positives and false negatives. Table 8 summarizes the resulting detection statistics.

As shown in Table 8, 96 of the 100 predictions were matched, with a mean matched IoU of 0.897. On these 96 pairs, pixel-level MAE, RMSE, Abs Rel, and δ<1.25 were 0.792 m, 1.173 m, 0.084, and 0.966, respectively. The matched target-level MAE, RMSE, and Abs Rel were 0.454 m, 0.566 m, and 0.034. The target-level RMSE changed from 0.564 to 0.566 m after excluding the four unmatched predictions, demonstrating stable target-depth performance under class-consistent GT matching.

### 4.7. Extraction-Strategy Analysis

Table 9 compares complementary calibration and extraction configurations. Together with the isolated loss-support experiment in Table 6, it characterizes both the end-to-end pipeline choices and the contribution of RoI-focused supervision.

Applying class-aware KDE to AdaBins yielded a target-level RMSE of 1.539 m, whereas applying KDE to the per-image test-GT affine diagnostic yielded 0.996 m. The distributions in Figure 5 illustrate the predictor-specific mode structures encountered by the common extraction procedure, while Table 6 independently verifies the benefit of RoI-focused supervision.

For the proposed calibration, both KDE variants yielded lower RMSE than center-crop extraction (0.566/0.564 versus 0.714 m), supporting the use of mode-based rather than fixed-spatial extraction for these RoIs. Class-agnostic KDE achieved a lower MAE (0.435 versus 0.455 m), while class-aware KDE achieved a marginally lower RMSE (0.564 versus 0.566 m). The class-aware and class-agnostic variants therefore provide comparable performance with an MAE–RMSE trade-off: class-aware routing achieved the lowest RMSE, while class-agnostic routing achieved the lowest MAE.

We further tested Scott-bandwidth multipliers of 0.50, 0.75, 1.00, 1.25, and 1.50; prominence thresholds of 0.01, 0.02, 0.03, and 0.05; and class-aware, highest-density, and nearest-depth routing. To make the full grid tractable while retaining all pixels, the sensitivity analysis used a 4096-bin weighted approximation of the Gaussian KDE. At the default setting, this approximation reproduced the exact proposed/AdaBins RMSEs as 0.5636/1.5391 m, compared with exact values of 0.5636/1.5389 m. Table 10 reports the corresponding one-factor analysis.

Across the complete 5×4×3 grid, the proposed method had a lower RMSE in 56 of 60 combinations. Under class-aware routing, it remained lower for every evaluated bandwidth multiplier and prominence threshold. This broad result demonstrates that the target-level advantage is stable across the principal bandwidth and prominence settings, while also quantifying the influence of the routing choice.

### 4.8. Qualitative Performance Evaluation and Visualization Analysis

**Figure 6:**
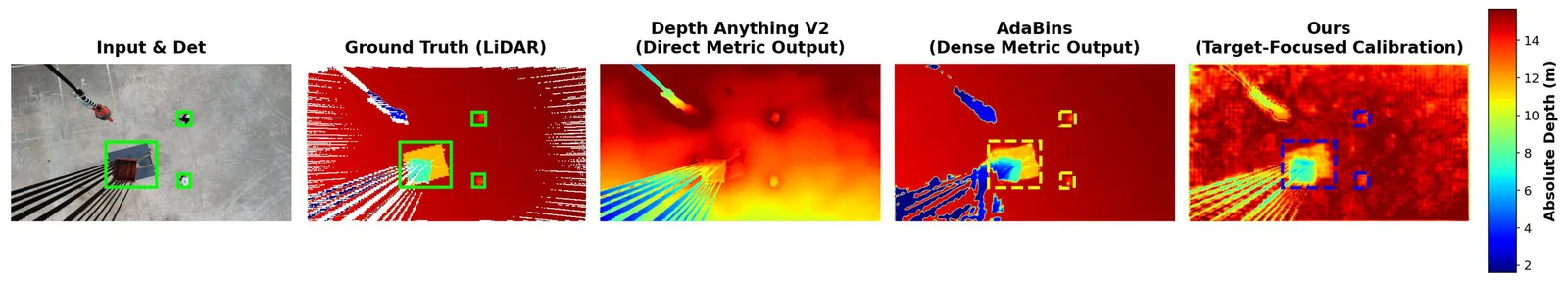
Representative qualitative depth comparison. Green boxes identify target regions in the input and LiDAR GT panels; yellow and blue dashed boxes identify the corresponding target regions in the AdaBins and proposed outputs, respectively.

Figure 6 provides representative qualitative outputs. Depth Anything V2 shows an absolute-scale mismatch, and the displayed AdaBins examples show smoother target–background transitions. The proposed output in the displayed examples retains sharper target transitions. The calibration map is predicted densely from foundation features and relative depth, while the BBox defines its GT-supervised training support rather than directly guiding the inference map.

**Figure 7:**
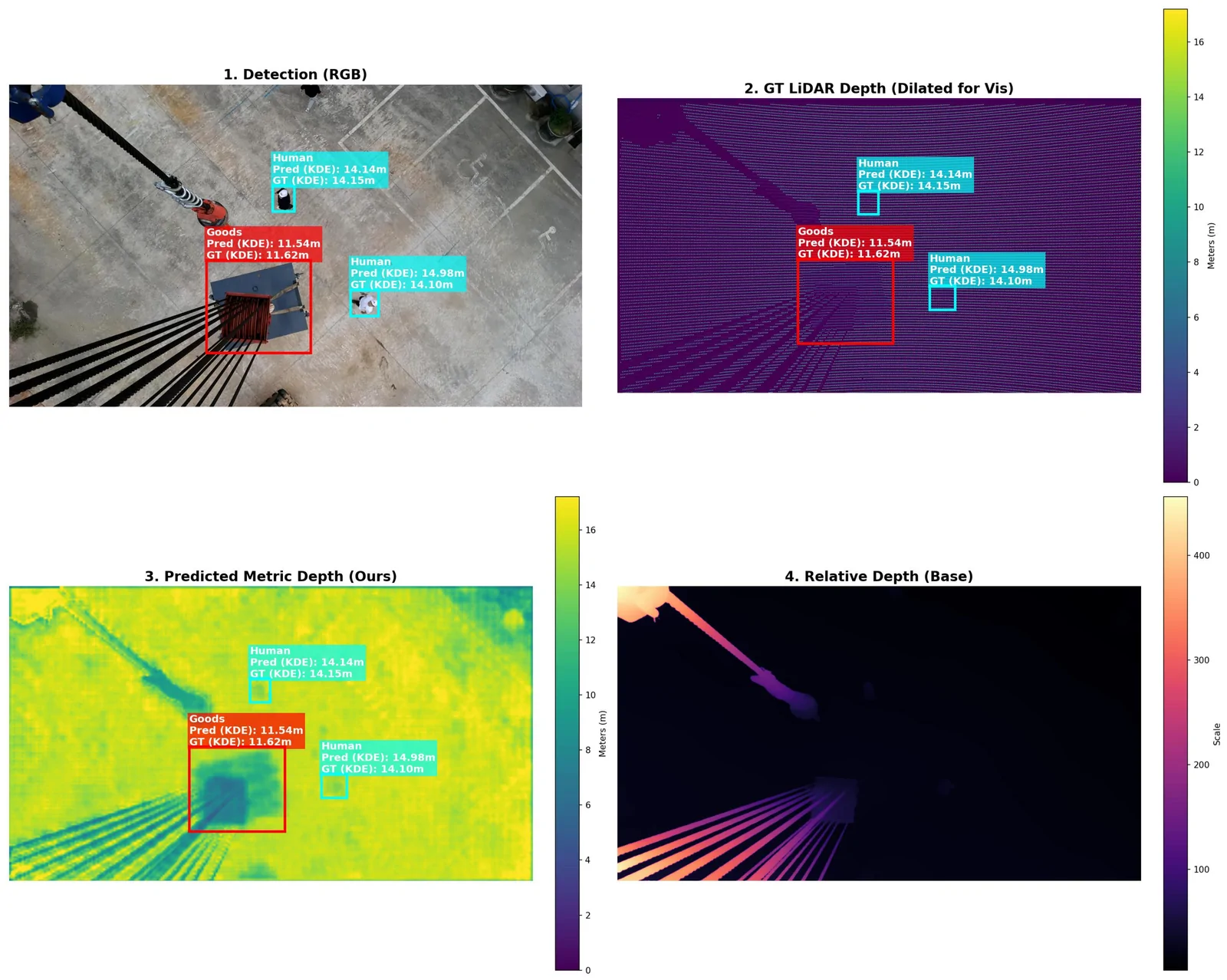
Simultaneous Goods and Human detections with dense calibrated depth and RoI-level distance estimates. LiDAR points are dilated only for visualization.

Figure 7 illustrates simultaneous detections at different camera-to-target distances within one frame. The detector supplies a class and BBox for each object, and the KDE stage extracts a separate representative distance from each corresponding region of the dense calibrated map, illustrating the intended multi-object pipeline output.

### 4.9. Computational Cost and Throughput

Table 11 reports component-wise and optimized complete-path latency at 896×896 using FP16 on one NVIDIA Titan RTX GPU. Component timings were synchronized and averaged over 20 runs after five warm-up runs. The optimized complete path was measured over all 27 test frames in three repetitions after one full-dataset warm-up pass (81 measurements); it includes one shared detection–calibration forward pass, original-resolution depth reconstruction, score filtering, and histogram-KDE extraction for every retained predicted RoI. Image acquisition, disk I/O, communication, and decision logic were excluded.

The proposed pipeline executes the fixed foundation once and shares the resulting features between the detection and calibration branches. For online inference, the class-aware density curve is computed using the validated 4096-bin weighted histogram approximation. Across the complete test set, the resulting compute-only path required 1.407±0.034 s/frame, corresponding to 0.71 FPS, with an average of 3.70 retained RoIs per frame. Exact Gaussian KDE is retained as an offline reference computation for validating the approximation rather than as the online extraction implementation.

For a nominal translational speed of 0.5–1.0 m/s, the measured 1.407 s update interval corresponds to approximately 0.70–1.41 m of travel. Slewing requires a geometry-specific analysis incorporating angular velocity, radius, and target position. The present implementation therefore supports low-rate online target ranging, while computational optimization and end-to-end timing evaluation under measured travel, payload, and slewing trajectories constitute the next steps toward real-time safety-alert deployment.

The histogram-KDE implementation reduced extraction latency from 647.0 to 13.9 ms per representative RoI while reproducing the default exact target RMSE within 0.0002 m. The remaining latency was dominated by the foundation and detection computation. Additional directions include replacing ViT-L with a smaller encoder, reducing input resolution subject to small-Human accuracy, structured pruning, knowledge distillation, quantization, and scheduling depth updates at a lower rate than detection or tracking. These alternatives motivate a dedicated accuracy–latency study under payload motion and slewing.

## 5. Discussion

The central empirical result is the complementary ranking obtained at the pixel and target levels. AdaBins achieved lower pixel-level MAE and RMSE, whereas the proposed pipeline achieved the lowest KDE-derived target-level errors and the highest pixel-level inlier ratios. This result confirms the importance of evaluating the representative object distance required by the crane-monitoring application in addition to dense-pixel averages. The isolated loss-support experiment further attributes a 0.151 m target-RMSE improvement to RoI-focused supervision under a controlled architecture and evaluation protocol.

Human RoIs constitute the most demanding target class because of their smaller average image area. Nevertheless, the proposed pipeline achieved lower Human and Goods target-level errors than AdaBins, supporting its use for both safety-relevant target categories. Higher-resolution small-target features and denser depth supervision provide promising directions for further improvement.

The frame-cluster confidence intervals, class-specific and tail statistics, isolated loss-support ablation, and straight-travel-to-slewing holdout provide complementary evidence for the proposed pipeline. In particular, the holdout evaluates all 125 slewing frames after training exclusively on straight-travel data and achieves target RMSEs of 0.915 m with GT RoIs and 1.175 m with predicted RoIs. The synchronized operational dataset and the physically distinct holdout condition extend the evaluation beyond a single aggregate test result. Future work will broaden this validation across additional crane installations, sensor poses, illumination, and weather conditions and will examine newer metric-depth models such as Metric3D v2 [31] and UniDepth V2 [32], temporal fusion, and end-to-end hazard-response timing.

## 6. Conclusions

This study presents an application-oriented sensing pipeline for target-focused metric-depth estimation in a top-view crane installation. A fixed DINOv2–DPT network supplies relative depth and backbone features, while the trainable multiscale adapter, DINO detection branch, and pixel-wise spatial affine calibration head provide object RoIs and dense metric predictions. During training, GT BBoxes restrict metric supervision to target-relevant RoIs.

Under a common predicted-RoI protocol, the proposed method achieved a pixel-level δ<1.25 ratio of 0.966 and target-level MAE/RMSE values of 0.455/0.564 m. A class-consistent one-to-one GT-matching analysis yielded 96 TP, 4 FP, and 3 FN, corresponding to precision/recall of 0.960/0.970. The matched target-level MAE/RMSE of 0.454/0.566 m differed from the common-RoI RMSE by only 0.002 m, confirming the stability of the target-depth result. AdaBins retained lower pixel-level MAE and RMSE in the common-RoI comparison, showing that dense-pixel and representative-target metrics measure different aspects of performance.

Mode-based KDE extraction yielded lower error than center-crop extraction for the proposed predictions, and the proposed method retained its target-level advantage across all evaluated class-aware bandwidth and prominence settings. Frame-cluster uncertainty analysis and the complementary straight-travel-to-slewing holdout further support the target-depth result beyond the primary aggregate comparison. Overall, the results demonstrate the feasibility and practical value of the proposed complementary camera-ranging pipeline for the evaluated top-view crane environment. Future work will extend validation to additional sites, sensor configurations, environmental conditions, temporal fusion, and optimized end-to-end execution.

## Figures and Tables

**Table 1:** Layer-level specification of the proposed pipeline at 896×896 input resolution.

Module	Input	Operation	Output	Status
DINOv2 ViT-L/14	RGB image	Patch embedding and transformer blocks	1024 channels, 64×64	Fixed
DPT decoder	Multilevel ViT features	Pretrained DPT decoder	1-channel relative-depth map	Fixed
Feature projection	Top ViT feature	1×1 conv, GN(32), GELU	256 channels, 64×64	Trainable
Adapter level 1	Projected feature	Two 2×2, stride-2 transposed convs; intermediate GN(32), GELU	256 channels, 256×256	Trainable
Adapter level 2	Projected feature	2×2, stride-2 transposed conv	256 channels, 128×128	Trainable
Adapter levels 3/4	Projected feature	Identity/2×2, stride-2 max pool	256 channels, 64×64/32×32	Shared trainable
ChannelMapper	Four adapter levels	1×1 conv, GN(32)	Four 256-channel levels	Trainable
DINO branch	Four mapped levels	6-layer encoder/decoder, 900 queries, 256-d embeddings, 8-head decoder self-attention	Classes and BBoxes	Trainable
Affine head	Feature and relative depth	3×3 conv (257–128), ReLU, 1×1 conv (128–2)	2 channels, 256×256	Trainable

**Table 2:** Statistics of the 150-ton crawler crane dataset.

Dataset Attribute	Specification/Statistics
Total frames	272 (train: 190, val: 55, test: 27)
Annotated instances	989 (train: 687, val: 203, test: 99)
Target classes	Human (ground personnel),
	Goods (suspended payload)
Camera installation height	15.24–16.49 m
Avg. BBox size (Goods, 4K)	685×650 pixels
Avg. BBox size (Human, 4K)	200×180 pixels
Operating conditions	Straight travel: 147 frames,
	Slewing: 125 frames
Data characteristics	Operational 150-ton crane,
	hardware-synchronized 4K RGB–LiDAR

**Table 3:** Training details for the supervised metric-depth baselines.

(**a**) Model and Input Configuration					
Method	Initialization		Trainable Part	Input Resolution	Batch Size
AdaBins	Crane-domain checkpoint		Complete network	768×384	4
ZoeDepth-N	Crane-domain checkpoint		Metric head; relative-depth core fixed	768×384	2
(**b**) Optimization Configuration					
Method	Optimizer	Learning Rate	Weight Decay	Loss	Epochs
AdaBins	AdamW	$10^{-5}$	0.01	Valid-pixel L1	20
ZoeDepth-N	AdamW	$10^{-5}$	0.01	Valid-pixel L1	20

**Table 4:** Pixel- and target-level depth performance within common predicted target RoIs. Downward and upward arrows indicate that lower and higher values are better, respectively; bold values indicate the best performance for each metric.

(**a**) Pixel-Level Metrics							
**Method**	**Type**	**MAE (m) ↓**	**RMSE (m) ↓**	**Abs Rel ↓**	δ<1.25↑	$\delta <1.25^{2}↑$	$\delta <1.25^{3}↑$
Depth Anything 3	Zero-shot	10.373	10.519	0.750	0.003	0.009	0.014
Depth Anything V2	Zero-shot	1.559	1.886	0.150	0.862	0.962	0.981
Per-image affine (test GT)	Diagnostic	1.262	1.657	0.145	0.923	0.953	0.980
ZoeDepth	Supervised (E2E)	0.931	1.302	0.107	0.931	0.970	0.980
AdaBins	Supervised (E2E)	**0.604**	**1.151**	**0.072**	0.952	0.973	0.982
**Proposed (target-aware)**	**Decoupled**	0.796	1.185	0.086	**0.966**	**0.979**	**0.984**
(**b**) Target-Level Metrics Using Class-Aware KDE							
**Method**		**MAE (m) ↓**		**RMSE (m) ↓**		**Abs Rel ↓**	
Depth Anything 3		9.844		9.887		0.750	
Depth Anything V2		1.361		1.621		0.105	
Per-image affine (test GT)		0.844		0.996		0.066	
ZoeDepth		1.314		1.750		0.101	
AdaBins		1.105		1.539		0.086	
**Proposed (target-aware)**		**0.455**		**0.564**		**0.034**	

**Table 5:** Target-level uncertainty, class-specific errors, and tail errors on the reported test split.

Method	Subset	*n*	MAE (m)	RMSE (m)	RMSE 95% CI (m)	P95 AE (m)	Max AE (m)
AdaBins	All	100	1.105	1.539	[1.265, 1.789]	3.954	4.052
Proposed	All	100	0.455	0.564	[0.501, 0.625]	1.058	1.496
AdaBins	Human	73	0.951	1.041	–	1.439	1.496
Proposed	Human	73	0.565	0.646	–	1.120	1.496
AdaBins	Goods	27	1.520	2.417	–	4.026	4.052
Proposed	Goods	27	0.156	0.217	–	0.481	0.664

**Table 6:** Isolated comparison of GT-BBox and complete-image metric-loss support under fixed predicted RoIs. Downward and upward arrows indicate that lower and higher values are better, respectively; bold values indicate the best performance for each metric.

(**a**) Pixel-Level Metrics						
**Metric-Loss Support**	**MAE (m)**	**RMSE (m)**	**Abs Rel**	δ<1.25	$\delta <1.25^{2}$	$\delta <1.25^{3}$
All valid LiDAR pixels	1.172	1.611	0.115	0.937	0.977	0.982
GT-BBox valid LiDAR pixels	**0.796**	**1.185**	**0.086**	**0.966**	**0.979**	**0.984**
(**b**) Target-Level Metrics Using Class-Aware KDE						
**Metric-Loss Support**	**MAE (m)**		**RMSE (m)**		**Abs Rel**	
All valid LiDAR pixels	0.519		0.715		0.040	
GT-BBox valid LiDAR pixels	**0.455**		**0.564**		**0.034**	

**Table 7:** Straight-travel-to-slewing holdout results on 125 slewing frames.

Evaluation	RoIs	Pixel RMSE (m)	Target MAE (m)	Target RMSE (m)	RMSE 95% CI (m)	Detector mAP/AP50
GT-RoI depth only	394	1.546	0.709	0.915	[0.830, 1.003]	–
Predicted-RoI pipeline	520	1.603	0.776	1.175	[0.962, 1.403]	0.610/0.915

**Table 8:** Class-consistent one-to-one GT-matching results at IoU ≥0.5.

Class	TP	FP	FN	Precision	Recall
Goods	27	0	0	1.000	1.000
Human	69	4	3	0.945	0.958
Overall	96	4	3	0.960	0.970

**Table 9:** Target-level performance of calibration and extraction strategies. The downward arrow indicates that lower values are better; bold values indicate the best performance for each metric.

Calibration and Extraction Strategy	MAE (m) ↓	RMSE (m) ↓
AdaBins (E2E) + KDE peak (class-aware)	1.105	1.539
Per-image affine (test GT) + KDE peak	0.844	0.996
Proposed (target-aware) + center-crop 50%	0.562	0.714
Proposed (target-aware) + KDE peak (class-agnostic)	**0.435**	0.566
**Proposed (target-aware) + KDE Peak (class-aware)**	0.455	**0.564**

**Table 10:** One-factor KDE sensitivity in target-level RMSE (m).

Varied Parameter	Value	Proposed	AdaBins
Bandwidth × Scott	0.50/0.75/1.00	0.538/0.579/0.564	1.582/1.567/1.539
	1.25/1.50	0.716/1.664	1.612/1.846
Prominence	0.01/0.02	0.562/0.564	2.082/1.539
	0.03/0.05	0.605/0.624	1.014/0.964
Routing	Class-aware	0.564	1.539
	Highest-density	1.174	1.408
	Nearest-depth	0.535	1.457

**Table 11:** Component-wise inference latency at 896×896 resolution.

Component or Execution Path	Latency (ms)	Device
Fixed feature and relative-depth extraction	965.0	GPU
Detection-branch remainder after shared feature extraction	277.3	GPU
Affine calibration and resizing	2.1	GPU
Shared detection–calibration forward pass	1311.7	GPU
Exact Gaussian KDE, per RoI	647.0	CPU
Histogram-KDE, per RoI	13.9	CPU
Optimized complete path with histogram-KDE	1406.8	GPU + CPU

## Data Availability

Upon publication, the authors will consider releasing an anonymized subset of the synchronized RGB–LiDAR data, the reported model weights, and the evaluation code for common-RoI metrics, KDE extraction, bootstrap intervals, and the operating-condition split, subject to applicable data-sharing and institutional requirements.
